# Non-Linear Optical Effects in Nanomaterials

**DOI:** 10.3390/nano9121717

**Published:** 2019-12-02

**Authors:** Jaroslaw Mysliwiec

**Affiliations:** Advanced Materials Engineering and Modelling Group, Wroclaw University of Science and Technology, 50-370 Wroclaw, Poland; jaroslaw.mysliwiec@pwr.edu.pl

Nonlinear optics is the domain of optics that studies the physical interaction between one or multiple optical beams of high intensity and an optical medium. One of the main topics in modern materials science and in the field of photonics is the search for materials that exhibit multiple useful properties, including large nonlinear optical (NLO) effects, making them suitable for applications in numerous multidisciplinary areas such as frequency conversion, lasing, multiphoton fluorescence microscopy, saturable absorption, or light switching. This Special Issue of *Nanomaterials*, including 11 original research works [1,2,3,4,5,6,7,8,9,10,11], presents a balanced view of the current state-of-the-art and recent advances in the field of second and third order NLO properties of materials on a nano- and microscale, including experimental as well as theoretical contributions.

A saturable absorber (SA) is one the most important device in a mode-locked fiber laser, which can emit ultra-short pulses. Using the SA with high nonlinearity in the fiber laser cavity, multi-wavelength pulses or optical modulation of ultrashort pulse generation can be achieved [1,2,3,5,6,8]. Another interesting topic is an optical limiting based on materials which are capable of exhibiting significant promise for laser protection because of their nonlinear optical properties [4]. The demand for low-damage threshold laser protection and high-sensitivity systems such as fragile human eyes has attracted a lot of attention. Nowadays, a great number of researchers pay attention to the metal complexes that represent promising candidates for nonlinear optics [9]. Emergent research activity has been also dedicated to investigate semiconductor-based nanostructures, such as quantum dots. The reported results could serve as a roadmap for practical design and implementation of far IR optical and optoelectronic devices [10].

In this Special Issue, we have also included a manuscript devoted to the relatively new area of nanoscience: nonlinear plasmonics and utilization of metal nanoparticles in the enhancement of fluorescence emission [11]. Finally, the last published article gives some insight into mechanisms responsible for photoinduced mass transport in functionalized azo-polymers [7]. A simple continuous time random walk model of surface relief grating (SRG) inscription reproduces the light-driven mass transport found in experiments as well as the fine structure of SRG.

I would like to thank all authors of this Special Issue of *Nanomaterials* for their contributions and the prompt and constructive comments from the reviewers, and also the consistently outstanding and efficient support from the editorial office.
